# Gingival Thickness Assessment at Mandibular Incisors of Orthodontic Patients with Ultrasound and Cone-beam CT. A Cross-sectional Study

**DOI:** 10.3290/j.ohpd.b1248965

**Published:** 2021-04-22

**Authors:** Dimitrios Kloukos, Lydia Kakali, George Koukos, Anton Sculean, Andreas Stavropoulos, Christos Katsaros

**Affiliations:** a Lecturer, Department of Orthodontics and Dentofacial Orthopedics, School of Dental Medicine, University of Bern, Bern, Switzerland; Department of Orthodontics and Dentofacial Orthopedics, 251 Hellenic Air Force & VA General Hospital, Athens, Greece. Performed all clinical and radiographic measurements, wrote the manuscript and prepared all figures, reviewed the manuscript.; b Researcher, Department of Orthodontics and Dentofacial Orthopedics, 251 Hellenic Air Force & VA General Hospital, Athens, Greece. Performed all clinical and radiographic measurements, wrote the manuscript and prepared all figures, reviewed the manuscript.; c Researcher, Department of Periodontology, 251 Hellenic Air Force & VA General Hospital, Athens, Greece. Performed all clinical and radiographic measurements, assisted with the interpretation of statistics, reviewed the manuscript.; d Professor and Chairman, Department of Periodontology, School of Dental Medicine, University of Bern, Bern, Switzerland. Oversaw the project and assisted with the writing of the manuscript, reviewed the manuscript.; e Professor, Department of Periodontology, Faculty of Odontology, Malmö University, Malmö, Sweden; Professor, Division of Regenerative Dental Medicine and Periodontology, CUMD, University of Geneva, Geneva, Switzerland. Wrote the manuscript and prepared all figures, reviewed the manuscript.; f Professor and Chairman, Department of Orthodontics and Dentofacial Orthopedics, School of Dental Medicine, University of Bern, Bern, Switzerland. Initiated and oversaw the project, assisted with the writing of the manuscript, reviewed the manuscript.

**Keywords:** cone-beam CT, gingival phenotype, periodontal tissue, ultrasound

## Abstract

**Purpose:**

To use and evaluate two methods for measuring gingival thickness (GT) at mandibular incisors of orthodontic patients and compare their performance in assessing periodontal anatomy through soft tissue thickness.

**Materials and Methods:**

The sample consisted of 40 consecutive adult orthodontic patients. GT was measured just before bracket placement at both central mandibular incisors, mid-facially on the buccal aspect, 2 mm apically to the free gingival margin with two methods: clinically with an ultrasound device (USD) and radiographically with cone-beam computed tomography (CBCT).

**Results:**

CBCT measurements were consistently higher than USD measurements, with the difference ranging from 0.13 mm to 0.21 mm. No statistically significant difference was noted between the repeated CBCT measurements at the right central incisor (bias = 0.05 mm; 95% CI = -0.01, 0.11; p = 0.104). Although the respective results for the left incisor statistically indicated that the measurements were not exactly replicated, the magnitude of the point estimate was small and not clinically significant (bias = 0.06 mm; 95% CI = 0.01, 0.11; p = 0.014). Small differences between CBCT measurements made by the 2 examiners at the left central incisor (bias = 0.06 mm; 95% CI = 0.01, 0.11; p = 0.014) were detected. However, this difference was minor and also not clinically significant. The respective analysis on the right incisor showed no statistically significant difference (bias = 0.05 mm; 95% CI = -0.01, 0.11; p = 0.246).

**Conclusions:**

Based on reproducibility, CBCT imaging for gingival thickness assessment proved to be as reliable as ultrasound determination. However, CBCT consistently yielded higher values, albeit at a marginal level, than did the ultrasound device.

The assessment of gingival thickness (GT) is often an important element that should be taken into consideration during treatment planning and subsequent decision making before various dental treatments. Quantitative and qualitative analysis of several periodontal parameters plays an important role, not only in planning periodontal procedures,^10,16,19^ but also in conventional prosthodontics,^24^ implant therapy,^21,25,27^ and orthodontics, when a change of tooth inclination is anticipated.^7,36^

Several methods have been recommended for measuring GT. Visual appraisal of gingival phenotype may be considered as relatively uncomplicated and time-saving. However, it might not always be considered an objective method: it has been demonstrated that, irrespective of the clinician’s skill, gingival phenotype is accurately identified in only about 50% of the cases.^14^ Another straightforward and commonly used method is transgingival probing with a periodontal probe.^23^ Potential limitations of this technique include the angulation of probe insertion, and the invasive nature of the procedure. Local anaesthesia is often required, which, in turn, has a two-fold limitation: patient discomfort and a transient local volume increase from injecting the local anaesthetic solution.^33^ Transgingival probing with an endodontic file has been proposed to overcome patient discomfort; nevertheless, problems with this method’s accuracy have been reported.^22^

An ultrasound device (USD) was proposed to resolve these limitations.^13^ The reproducibility of this method has been reported to be high.^27^ Another routinely applied procedure to classify gingival phenotype as thin or thick involves placement of a periodontal probe in the gingival sulcus; then, its transparency through the soft tissue is appraised.^20^ This method has also been reported to be highly reproducible, with 85% agreement between duplicate measurements.^12^

Finally, the use of cone-beam computed tomography (CBCT) has also shown a high diagnostic accuracy in assessing GT, demonstrating minimal discrepancy to clinical and radiographic measurements.^4,16,17^ A few studies have suggested CBCT as a standard method for determining gingival and bone thickness.^1,8,16,29^ Recently, technological developments have resulted in CBCT devices with lower radiation emissions,^9^ which renders it applicable in almost all dental procedures, although each patient should be evaluated individually based on their unique treatment needs and set of circumstances.

Nevertheless, it is still unclear which is the most suitable method for assessing GT. The lack of a gold standard technique to assess GT in human studies does not permit any clear recommendations for the clinician.

Since limited evidence-based data exist to validate the accuracy of CBCT in evaluating the thickness of soft tissues in the oral cavity, the aim of the present study was to compare CBCT with USD and determine the comparability and applicability of these methods as diagnostic tools for assessing GT on a subset of patients from a larger prospective study on orthodontic treatment, GT and the development of gingival recessions.

## Materials and Methods

### Ethics Approval and Consent to Participate

All procedures were in accordance with the ethical standards of the institutional and national research committee and with the 1964 Helsinki declaration and its later amendments. The study protocol was approved by the 251 Greek Air Force Hospital’s Education, Ethics and Research Committee (approval number: 076/7592/06.05.2015). All patients, or their legal guardian, provided written consent to participate prior to any measurements or performance of CBCT.

### Sample Selection

This cross-sectional study clinically evaluated GT in 40 white (Caucasian; ≥ 16 years old) orthodontic patients just before the commencement of orthodontic treatment, who consecutively visited the Department of Orthodontics and Dentofacial Orthopedics, 251 Hellenic Airforce Hospital, Athens, Greece.

CBCTs were not performed primarily for evaluating GT; CBCTs were principally carried out in order to assess bone condition and turnover in the mandibular anterior region, as part of an ongoing prospective controlled study assessing the occurrence of gingival recessions in orthodontically treated patients.

Exclusion criteria were: presence of crown restorations or fillings involving the cervical part of the anterior mandibular teeth, pregnant or lactating females, presence of obvious clinical signs of gingival conditions/diseases resulting in swelling of the gingiva (e.g. gingivitis), or presence of increased probing depths (e.g. > 3 mm) at the mandibular central incisors, presence of labial gingival recessions at the mandibular central incisors, intake of medication with any known effect on the gingiva, (e.g. calcium antagonists, etc.), presence of congenital anomalies or dental structural disorders.

### Sample Size Calculation

Although this cohort of patients derived from another ongoing, prospective study assessing gingival recessions in orthodontically treated patients, a current sample size calculation was performed using the formula of comparing two means, and included 90% power and statistical significance of 0.05. The standard deviation applied was 0.18 mm according to previous research^22^ and the anticipated mean difference was 0.20 mm.

### Clinical and Measuring Parameters

All clinical procedures, as well as CBCT imaging, were performed before bracket placement. Measurements were carried out at both central mandibular incisors, mid-facially on the buccal aspect of each tooth, and 2 mm apical to the free gingival margin, with the following two methods.

#### Ultrasound

**Fig 1:**
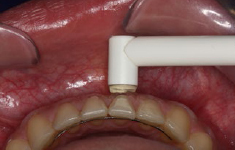
Measurement procedure with the ultrasound device.

A periodontist (GK) assessed the GT of each patient with the ultrasound device (USD; Krupp SDM, Austenal Medizintechnik; Cologne, Germany). Measuring GT with USD is based on the ultrasonic pulse-echo principle: ultrasonic pulses are transmitted through the sound-permeable tissue (1518 m/s) and are reflected at the surface of the hard tissue. By timing the received echo, GT is determined and digitally displayed. Measurements may range between 0.5 and 8.0 mm with a resolution of 0.1 mm. Ultrasonic frequency is 5 MHz and the diameter of the transducer probe is 3 mm with a weight of 19 g. Measurements were performed by perpendicularly placing the transducer probe on the gingival surface without pressure, ensuring that the center of the transducer was 2 mm apical to the free gingival margin (Fig 1).

#### CBCT imaging

All patients underwent CBCT examination in a private clinic (Orofacial Radiodiagnosis; Athens, Greece). CBCT images were acquired using the Morita Accuitomo 80 3D Imaging System (J. Morita; Darmstadt, Germany) at 90 kV and 7 mA for 17.5 s and a single 360-degree image rotation. The CBCT scans were obtained with 6 x 6 cm field of view and 80 µ voxel size. Images were processed by I-Dixel-3DX software, v 2.0 (J. Morita; Darmstadt, Germany). During the examination, a cotton roll was used to retract the lip and enable the imaging of labial soft tissues.

### GT Measurement using CBCT Imaging

The GT in all CBCT images was measured independently by two authors (DK, LK) and recorded in data extraction forms without patient identification information. The first examiner (DK) conducted the measurements twice with an intermediate interval of one month in order to evaluate the intra-examiner repeatability.

**Fig 2:**
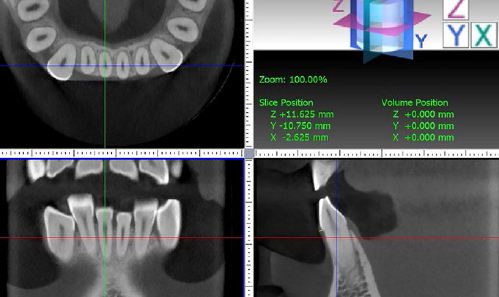
Measurement procedure in the CBCT images.

The method for measuring GT in the software was standardised after calibration between the two assessors using ten randomly selected CBCTs. This was done to ensure reproducibility of the measurement location (2 mm apical to the free gingival margin), as was the case for the clinical measurements with the ultrasound transducer probe. Measurements in the CBCT images were then performed perpendicularly to the tooth axis (Fig 2).

### Statistical Analysis

Descriptive statistics were performed for age, CBCT and USD measurements of gingival thickness. The repeated CBCT measurements by the first examiner were tested for systematic differences (bias) using paired t-tests. Repeatability was quantified via the 95% repeatability coefficient.^5,6^ The presence of a magnitude-related trend for the differences as well as for their dispersion was assessed graphically. Additionally, the presence of a trend for the differences was assessed statistically using Spearman’s rank correlation coefficient. The normality assumption was examined both graphically and via the Shapiro-Wilk test. The agreement between the two examiners on GT measurements from CBCT data was assessed both statistically and graphically. Paired t-tests were applied to test for systematic differences between the two examiners, while the reproducibility was quantified via the 95% reproducibility coefficient and in accordance with the repeatability coefficient. Again, normality assumptions and magnitude-related trends were evaluated as above. Method agreement was evaluated between CBCT and USD measurements using two separate Bland-Altman analyses. Finally, the 95% Limits of Agreement (95% LOA) and the corresponding 95% CIs were calculated. Normality assumptions were evaluated graphically and by means of the Shapiro-Wilk test. Statistical significance was set to α = 5%. All statistical analyses and graphical plots were conducted or constructed, respectively, using Stata 13.0/SE software (StataCorp; College Station, TX, USA).

## Results

Forty subjects (17 females and 23 males) participated in this study. The descriptive statistics for age, CBCT and USD measurements are reported in Table 1.

**Table 1:** Descriptive statistics of all gingival thickness measurements (in mm)

	Mean (SD)	Min	Max
Age	24.48 (6.68)	18.00	45.00
**Mandibular left central incisor**			
CBCT: Examiner #1	0.93 (0.24)	0.55	1.51
CBCT: Examiner #2	1.01 (0.24)	0.61	1.59
USD	0.80 (0.26)	0.50	1.50
**Mandibular right central incisor**			
CBCT: Examiner #1	0.95 (0.27)	0.50	1.50
CBCT: Examiner #2	0.99 (0.25)	0.42	1.43
USD	0.80 (0.22)	0.50	1.50

CBCT: Cone-beam computed tomography; USD: ultrasound device.

### Repeatability Assessment

The results of the paired t-tests for bias between the 1st and the 2nd CBCT measurements made by the first examiner (DK) are reported in Table 2. Repeatability of USD measurements were performed in a previous cross-sectional study with the same methodology and objective.^22^ The normality assumption was not violated for any of the differences between the repeated measurements of USD at two time points (mean difference 0.00; 95% CI -0.05, 0.05; p = 1.00).

**Table 2:** Results of the paired t-tests between the repeated CBCT measurements by examiner #1

Tooth	Bias (SE)	95% CI	p-value
Mandibular left central incisor	0.06 (0.02)	(0.01, 0.11)	0.014
Mandibular right central incisor	0.05 (0.03)	(-0.01, 0.11)	0.104
Bias = 2nd 1st measurements (in mm)			

SE: standard error.

Statistical analysis indicated that the repeated CBCT measurements were not identical for the mandibular left central incisor (bias = 0.06 mm; 95% CI = 0.01, 0.11; p = 0.014), whereas the repeated CBCT measurements for the mandibular right central incisor could be considered identical (bias = 0.05 mm; 95% CI= -0.01, 0.11; p = 0.104). Nevertheless, a difference of 0.06 mm in repeated measurements can be generally regarded as clinically irrelevant. The corresponding Bland-Altman plots are displayed in Fig 3a and 3b. Neither a magnitude- nor a dispersion-related trend could be identified graphically. An absence of a magnitude trend was also implied by the Spearman’s rank correlation coefficient (mandibular left central incisor: Spearman’s rho = 0.10, p-value= 0.554; mandibular right central incisor: Spearman’s rho = -0.14, p = 0.377). The Shapiro-Wilk test results showed that the normality hypothesis was valid for both left and right mandibular incisors (p = 0.595 and 0.614, respectively).

**Fig 3a:**
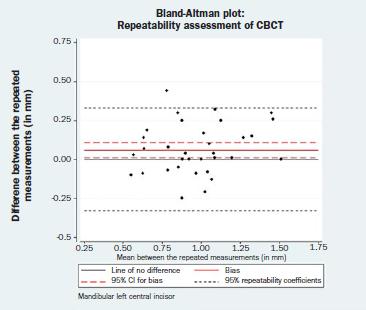
Bland-Altman plot: repeatability assessment for CBCT measurements, mandibular left central incisor.

**Fig 3b:**
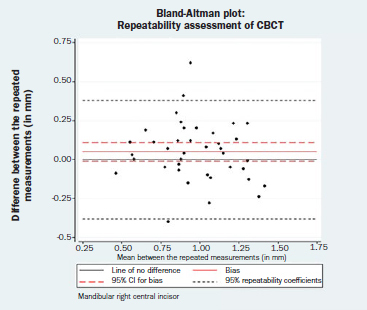
Bland-Altman plot: repeatability assessment for CBCT measurements, mandibular right central incisor.

### Reproducibility Assessment

The results of the paired t-tests for bias between the two examiners (DK, LK) are reported in Table 3. Again, statistical analysis indicated that the repeated measurements were not identical for the mandibular left central incisor (bias = 0.06 mm; 95% CI= 0.01, 0.11; p = 0.014), whereas the repeated measurements for the mandibular right central incisor could be considered identical (bias = 0.05 mm; 95% CI= -0.01, 0.11; p = 0.246). Nevertheless, a difference of 0.06 mm in different operators’ measurements can be generally regarded as clinically irrelevant. The corresponding Bland-Altman plots are displayed in Figs 3c and 3d. Neither a magnitude- nor a dispersion-related trend was identified (mandibular left central incisor: Spearman’s rho = -0.03, p = 0.836; mandibular right central incisor: Spearman’s rho = -0.12, p = 0.377). The normality hypothesis was valid for both left and right mandibular central incisors (Shapiro-Wilk test p = 542 and 0.475, respectively).

**Fig 3c:**
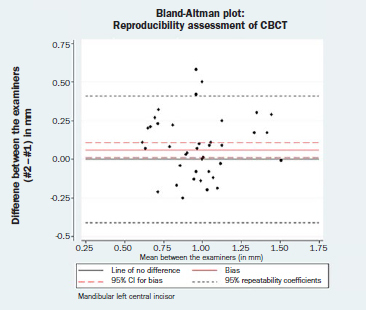
Bland-Altman plot: reproducibility assessment for CBCT measurements, mandibular left central incisor.

**Fig 3d:**
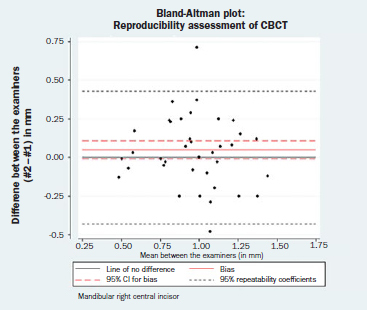
Bland-Altman plot: reproducibility assessment for CBCT measurements, mandibular right central incisor.

**Table 3:** Results of the paired t-tests between the CBCT measurements made by the two examiners

Tooth	Bias (SE)	95% CI	p-value
Mandibular left central incisor	0.06 (0.02)	(0.01, 0.11)	0.014
Mandibular right central incisor	0.05 (0.03)	(-0.01, 0.11)	0.246
Bias = examiner #2 – examiner #1 (in mm)			

SE: standard error.

### Method Agreement (Comparability)

The results of the paired t-tests between the two GT measuring techniques as well as the estimated corresponding 95% LOA and the respective 95% CIs are reported in Table 4. The respective Bland-Altman plots are displayed in Figs 4a to 4d. There was no evidence of a magnitude-related trend for either the differences or their dispersion after graphical evaluation.

**Fig 4a:**
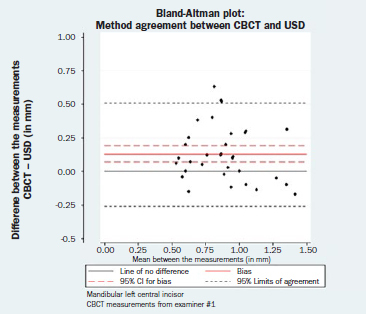
Bland-Altman plot: method agreement. Examiner #1, mandibular left central incisor.

**Fig 4b:**
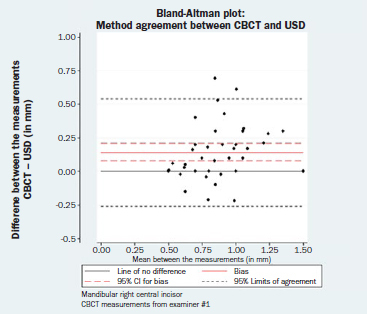
Bland-Altman plot: method agreement. Examiner #1, mandibular right central incisor.

**Fig 4c:**
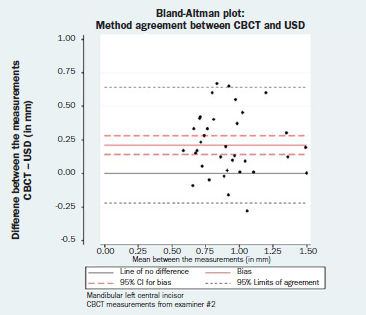
Bland-Altman plot: method agreement. Examiner #2, mandibular left central incisor.

**Fig 4d:**
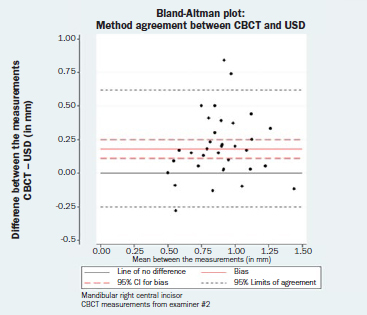
Bland-Altman plot: method agreement. Examiner #2, mandibular right central incisor.

**Table 4:** Results of the paired t-tests (bias, SE, 95% CI, p-value) between the CBCT and USD measurements, as well as the corresponding 95% LOA and the respective 95% CI for the LOA

	Bias (SE)	95% CI for bias	p-value	95% LOA	95% CI for the LOA	
					Lower LOA	Upper LOA
**Mandibular left central incisor**						
CBCT measurements from examiner #1	0.13 (0.03)	(0.07, 0.19)	<0.001	(-0.26, 0.51)	(-0.35, -0.17)	(0.42, 0.60)
CBCT measurements from examiner #2	0.21 (0.04)	(0.14, 0.28)	<0.001	(-0.22, 0.64)	(-0.32, -0.12)	(0.54, 0.75)
**Mandibular right central incisor**						
CBCT measurements from examiner #1	0.14 (0.03)	(0.08, 0.21)	<0.001	(-0.26, 0.54)	(-0.35, -0.16)	(0.45, 0.63)
CBCT measurements from examiner #2	0.18 (0.04)	(0.11, 0.25)	<0.001	(-0.25, 0.62)	(-0.36, -0.15)	(0.52, 0.72)
Bias = CBCT – USD (in mm).						

CBCT: cone-beam computed tomography; CI: confidence intervald; LOA: limit of agreement; USD: ultrasound device.

Finally, none of the normality assumptions could be rejected after either graphical evaluation or using Shapiro-Wilk tests (mandibular left central incisor, CBCT measurements from examiner #1: p = 0.163; CBCT measurements from examiner #2: p = 0.561; mandibular right central incisor, CBCT measurements from examiner #1: p = 0.157; CBCT measurements from examiner #2: p = 0.097).

## Discussion

The objective of the present study was to assess GT with non-invasive methods. Mandibular incisors were in focus, since a change in their inclination or root torque may introduce a risk factor for gingival recessions, making this an area of major concern in both functional and aesthetic respects.

Direct measurement with either a periodontal probe or an endodontic file is regarded as a fairly objective method for GT assessment. However, since it involves tissue penetration, its clinical application is associated with some limitations.^37^ These are often linked with measurement errors, probably originating from instruments’ rounded tips and thickness.^22^

An USD showing high reproducibility^13,22,27,28^ was selected as the first non-invasive method for measuring GT. The second selected method was CBCT imaging, which that has been shown to have high diagnostic applicability.^4,16^

The present results show that the difference between USD and CBCT measurements of gingival thickness was not zero. CBCT measurements were constantly higher than those obtained with the USD, with the difference ranging from 0.13 mm to 0.21 mm (Table 4). This difference was independent of the magnitude of GT measurement. Although it is difficult to assign the difference reported to one methodology or the other, the difference could possibly be attributed to the ultrasound procedure, due to measuring imprecision such as misangulation of the ultrasound transducer or over-compression of the soft tissue.

There were no statistically significant differences between the repeated measurements made by the first examiner on the mandibular right central incisor (p = 0.104). Although the respective results for the mandibular left central incisor indicated that the measurements were not exactly true replicates from a statistical point of view, the magnitude of the point estimate of bias was small and possibly not clinically significant (bias = 0.06 mm; p = 0.014).

Moreover, there was evidence of a small systematic difference between the CBCT measurements made by the two examiners on the mandibular left central incisor (bias = 0.06 mm, 95% CI = 0.01, 0.11). However, this difference was minor, and again, clinically unimportant. On the other hand, the respective analysis on the mandibular right central incisor showed no statistically significant difference between the two examiners (bias = 0.05 mm, 95% CI= -0.01, 0.11).

Numerous dental procedures require accurate measurement of GT, since respect of the gingival phenotype is vital and appears to influence the outcomes of various treatment strategies. Gingival phenotype evaluation through simple visual appraisal is inaccurate,^11,14^ mostly due to its subjective nature; to a great extent, it relies on clinical competence. Thick gingiva, i.e. > 0.81 mm thick, is shown to be relatively resistant to gingival recession following surgical or restorative therapies,^2,3,15,31^ whereas thin-scalloped gingiva is considered at risk, because it has been associated with a compromised response following the same treatments.^2,2,15,25,30-32^ These findings point clearly to the need for a thorough diagnosis, through a straightforward and reproducible method, of these high-risk patients, prior to various interventions involving the gingiva. As far as accuracy is concerned, it is important to bear in mind that this term refers to the proximity of the measurements to the true value of gingival thickness. By definition, the true value cannot be measured by methods such as those in the present study. Only an estimation of the true value is possible. This is the reason why it is important to describe repeatability, reproducibility and the correlation of the methods tested.

Our study is not free of limitations, although efforts were made to minimise them. Firstly, the clinical measurements did not take into account potential differences in dental arch crowding or tooth inclination that may influence the clinical handling of the USD transducer probe, although it was expected that this would not lead to a large method error. Secondly, at present, conducting CBCT to assess GT might not be justified due to the amount of radiation, as CBCT involves higher doses than two-dimensional imaging. Moreover, CBCT images have a certain degree of inaccuracy, attributed primarily to image generation, processing, voxel size and various types of artefacts that might be present. In general, the smaller the voxel size, the higher the precision/resolution of the information provided. Larger voxels may include different tissues, so that the subsequent grayscale value may not clearly indicate one specific tissue, such as bone. This issue is primarily evident at the limits between neighbouring tissue types of different radiodensity. However, at the same time, the smaller the voxel size, the higher the motion artefacts. Thus, based on the above considerations and also on the need to keep radiation exposure as low as possible, a specific CBCT image can only reach a certain degree of detail of the information it provides.^18,34,35^

Finally, it has to be pointed out that the lack of a gold standard procedure for measuring soft tissue thickness may downgrade the clinical significance. However, finding a gold standard measurement for humans is almost impossible, because all clinical or imaging methods present an inherent measurement error which is not always easy to assess during implementation. On the other hand, the relevance of the current study lies in the fact that it includes both imaging and clinical procedures and provides robust data for the comparison of the tested methods.

## Conclusions

Based on reproducibility, CBCT imaging for gingival thickness assessment proved to be as reliable as ultrasound determination. However, CBCT imaging yielded consistently higher values, albeit at a marginal level, than did the ultrasound device.
